# SUZ12 is a novel putative oncogene promoting tumorigenesis in head and neck squamous cell carcinoma

**DOI:** 10.1111/jcmm.13638

**Published:** 2018-04-18

**Authors:** Yaping Wu, Huijun Hu, Wei Zhang, Zhongwu Li, Pengfei Diao, Dongmiao Wang, Wei Zhang, Yanling Wang, Jianrong Yang, Jie Cheng

**Affiliations:** ^1^ Jiangsu Key Laboratory of Oral Disease Nanjing Medical University Jiangsu China; ^2^ Department of Oral and Maxillofacial Surgery Affiliated Stomatological Hospital Nanjing Medical University Jiangsu China; ^3^ Department of Oral Pathology School of Stomatology Nanjing Medical University Jiangsu China

**Keywords:** biomarker, head and neck squamous cell carcinoma, polycomb repressive complex, SUZ12

## Abstract

The suppressor of zest 12 (SUZ12), one of the core polycomb repressive complex 2 (PRC2) components, has increasingly appreciated as a key mediator during human tumorigenesis. However, its expression pattern and oncogenic roles in head and neck squamous cell carcinoma (HNSCC) remain largely unexplored yet. Here, we sought to determine its expression pattern, clinicopathological significance and biological roles in HNSCC. Through data mining and interrogation from multiple publicly available databases, our bioinformatics analyses revealed that SUZ12 mRNA was significantly overexpressed in multiple HNSCC patient cohorts. Moreover, SUZ12 protein was markedly up‐regulated in primary HNSCC samples from our patient cohort as assessed by immunohistochemical staining and its overexpression significantly associated with cervical node metastasis and reduced overall and disease‐free survival. In the 4‐nitroquinoline 1‐oxide (4NQO)‐induced HNSCC mouse model, increased SUZ12 immunostaining was observed along with disease progression from epithelial hyperplasia to squamous cell carcinoma in tongue. Furthermore, shRNA‐mediated SUZ12 knock‐down significantly inhibited cell proliferation, migration and invasion in HNSCC cells, and resulted in compromised tumour growth in vivo. Collectively, our data reveal that SUZ12 might serve as a putative oncogene by promoting cell proliferation, migration and invasion, and also a novel biomarker with diagnostic and prognostic significance for HNSCC.

## 1 INTRODUCTION

Head and neck squamous cell carcinoma (HNSCC) represents the sixth most common malignancy worldwide with more than 600 000 new cases and 350 000 cancer‐related deaths reported annually.^1^ The well‐established aetiological risks for this malignancy include smoking, alcohol, betel quid chew and human papillomavirus (HPV) infection. Progress in HNSCC treatment has remarkably improved the quality of life and life expectancy of patients especially when disease is diagnosed at its early stage. However, the overall survival of patients with HNSCC, especially the majority of which is diagnosed at advanced stages, has not been substantially improved over the past decades.^2^ Multiple clinicopathological parameters including locoregional relapse, cervical lymph node metastasis and clinical stage at diagnosis have been recognized as the key factors affecting patient prognosis.^3^ Previous intensive efforts were made to unravel the genetic, epigenetic and environmental factors driving HNSCC tumorigenesis.^4^ However, in‐depth mechanistic understanding about HNSCC initiation and progression still remains far from complete. Thus, development of effective treatment for HNSCC will largely hinge on the identification of genetic alterations and relevant molecular mechanisms during HNSCC carcinogenesis to find druggable targets of therapeutic values.

Epigenetic abnormality has been increasingly recognized as a hallmark of cancer and significantly contributes to cancer initiation and progression.^5^ Until now, dozens of epigenetic modulators have been identified as pivotal mediators driving tumorigenesis and held great promise as therapeutic targets against cancer largely due to their pervasive roles as well as inherent reversible nature of epigenetic alternations.^6^ Among these cancer‐associated epigenetic modulators, polycomb group (PcG) proteins have stood out as essential participators of malignant transformation, metastatic spreading and promising targets for therapeutic intervention.^7^ Mounting evidence has established that these PcG components commonly assemble into protein complexes with additional factors which are recruited to specific chromatin regions to maintain target genes in a transcriptionally repressive state. Briefly, two canonical PcG complexes (PRC1 and PRC2) harbour multiple core members to execute their functions by histone modifications.^8^ As expected, our previous studies and others have provided strong evidence that multiple PcG members such as Bmi1 and EZH2 might function as bona fide oncogenes driving tumorigenesis in diverse sites and their elevated abundance associates with cancer aggressiveness and unfavourable prognosis in a myriad of human cancer including HNSCC.^7^, ^9^, ^10^, ^11^, ^12^, ^13^ Recently, another PcG member, suppressor of zest 12 (SUZ12), which is essential for PRC2‐mediated gene silencing by generating trimethylation on lysine 27 residue of histone H3 (H3K27me3), has been increasingly appreciated as a putative oncogene or tumour suppressor underlying tumorigenesis.^14^, ^15^, ^16^ SUZ12 has been found to be frequently overexpressed in several solid cancers including colorectal, ovarian and non‐small lung cancer. Its aberrant overexpression significantly associated with aggressive clinicopathological features and inferior survival.^14^, ^17^, ^18^ Furthermore, SUZ12 knock‐down induced impaired tumour growth, invasion and metastasis in bladder, gastric and lung cancer.^17^, ^19^, ^20^ However, on contrary, recurrent loss‐of‐function somatic alterations of SUZ12 have been identified in malignant peripheral nerve sheath tumour and contribute to its initiation and progression.^16^ Therefore, these data highlight the complexity of biological roles of SUZ12 underlying tumorigenesis in diverse contexts. However, to the best of our knowledge, the expression of SUZ12 and its clinicopathological significance in HNSCC have not been established yet.

In this study, we sought to investigate the expression of SUZ12 and its clinicopathological significance in primary human HNSCC samples and chemical‐induced animal model. The tumorigenic roles of SUZ12 in HNSCC were also further explored both in vitro and in vivo.

## 2 MATERIALS AND METHODS

A panel of HNSCC cell lines including Cal27, Fadu, SCC4, SCC25, HN4 and HN6 were used in this study. Normal human oral keratinocytes (HOK), Cal27, Fadu, SCC4 and SCC25 cells were purchased from American Type Culture Collection (ATCC) and authenticated by short tandem repeat (STR) profiling. HN4 and HN6 cells were kindly gifted from Dr. Wantao Chen (Shanghai Jiao Tong University). All cells were routinely tested for mycoplasma at regular intervals. Cells were grown in DMEM/F12 (Invitrogen) supplemented with 10% FBS (Gibco) and penicillin‐streptomycin (1%), and maintained at 37°C in a 5% CO2‐humidified incubator.

### 2.1 SUZ12 knock‐down by shRNA lentiviral vector

Two short hairpin RNAs (shRNA) against human SUZ12 were subcloned into pLKO.1 puro lentiviral plasmid and verified by direct sequencing. The sequences were listed as follows: shSUZ12‐1: GCTGACAATCAAATGAATCAT (TRCN0000038728); shSUZ12‐2: CCACAAGAAATGGAAGTAGAT (TRCN0000038724). The shRNA vector containing sequence without targeting any known human gene was used as negative control (shNC). Lentiviral particles were prepared by transiently cotransfecting HEK293T cells with lentiviral vectors together with the packaging and envelope plasmids (pCMV‐VSV‐G and pCMV‐Δ8.2) using calcium‐phosphate method. The viral supernatants were filtered and concentrated after 48‐72 hours, and then, these lentiviral particles were used to infect cells or stored at −80°C. The efficiencies of shSUZ12 lentiviral vectors were confirmed by Western blot following cell infection in vitro. The stable SUZ12 knock‐down cell clones were selected by appropriate antibiotics (puromycin, 2‐5 μg/mL, Sigma) for at least 1 week after virus infection.

### 2.2 Cell proliferation by MTT assay and apoptosis by flow‐cytometric assay

Cell proliferation and viability were determined by absorbance with MTT assay. Approximately 3000 cells per well were seeded in the 96‐well plates. At the indicated time‐points, 5 mg/mL MTT (Sigma) was added to the plates and incubated at 37°C for another 4 hours. Absorbance at 490 nm was measured with an automatic enzyme‐linked immunosorbent assay reader (BioTek Instruments). For cell apoptosis assay, cells were stained with Annexin V: PI Apoptosis Detection Kit (BD Bioscience) and processed using the BD FACSuite analysis software.

### 2.3 Cell migration and invasion assay

Cell migration and invasion assays in vitro were performed with wound‐healing and transwell chambers (8‐μm pore size, Corning) with Matrigel (BD Pharmingen) pre‐coating, respectively. For wound‐healing assay, cells were grown into monolayer and scratched using a sterile 200‐μL pipette. Cell migration was observed at various time‐points later by microscopy. Images of 10 scratches per cells under each experimental condition were captured at the same locations and then compared with ImageJ software (NIH). For cell invasion assay, Boyden chambers were pre‐coated with 100% Matrigel, and then, cells were seeded in the upper chambers with serum‐free medium. Complete growth medium containing 10% FBS in the lower chambers served as chemoattractant. The non‐invaded cells were gently removed with a cotton swab and those invaded cell located on the lower side were fixed and stained with crystal violet, and counted.

### 2.4 Immunofluorescence assay

For cell immunofluorescent staining, cells were pre‐seeded and grown on glass coverslips 24 h prior to experiment and then fixed with 4% paraformaldehyde and permeabilized in Triton X‐100 (0.1% in PBS) and sequentially blocked with 3% bovine serum albumin (BSA) for 30 minutes. Following the overnight incubation with primary antibody against SUZ12 (1:200 dilution), these cells were further incubated with secondary antibody and cytoskeleton actin/nuclear staining. Immunofluorescence was visualized under a Zeiss fluorescence microscope or confocal microscope, and then image captured using similar parameters.

### 2.5 Western blot analysis

Cells in culture flasks or plates were lysed in ice‐cold buffer containing protease inhibitor cocktail (Roche). Equal amounts of protein samples were loaded and separated by 8%‐12% SDS‐PAGE and transferred to PVDF membranes (Millipore) followed by 5% non‐fat milk or 3% BSA blocking. These blots were incubated at 4°C overnight with primary antibodies against SUZ12 (1:1000 dilution; Abcam, ab112073, USA), β‐actin (1:1000 dilution; Santa Cruz, sc‐47778, USA) and GAPDH (1:2000 dilution; Abcam, ab8245, USA) followed by incubations with the corresponding secondary antibodies. The relative levels of each protein were quantified with ImageJ software (NIH).

### 2.6 4‐nitroquinoline 1‐oxide (4NQO)‐induced HNSCC animal model

The 4NQO‐induced HNSCC animal model in which squamous cell carcinoma was initiated and maintained in tongue was performed as previous reports with minor modifications.^13^, ^21^, ^22^ For 4NQO‐induced tongue SCC in C57BL/6 mice, 6‐week‐old mice were treated with drinking water containing 50 μg/mL 4NQO for consecutive 16 weeks and then given with normal drinking water for another 8 weeks. Another group of animals with normal drinking water was used as negative control. The lesions in tongue were visually inspected every week. Samples were harvested at 16, 20 and 24 weeks after chemical administration and subjected to further histopathological analyses.

### 2.7 HNSCC xenograft model

All experiments involving animal subjects were in accordance with the institutional animal welfare guideline and approved by Institutional Animal Care and Use Committee of Nanjing Medical University. Six‐week‐old female nu/nu mice were obtained and maintained in a specific pathologic‐free environment. Stable SUZ12 knock‐down cancer cells (2 × 10^6^) suspended in total 100 μL PBS and Matrigel (1:1) were inoculated subcutaneously on the right flanks (6 animals per experimental group). Tumour growth was monitored after injection, and tumour diameters were measured by calipers every 3 days when tumour masses were identified. Tumour volume is calculated as follows: volume = a × b^2^/2, where a and b are defined as the longest diameter and shortest diameter, respectively. Final tumour weights were also measured upon animals were killed. The tumour samples were further processed for H&E staining, immunohistochemical staining, etc.

### 2.8 Data mining and analysis of SUZ12 in HNSCC via publicly available database

The original data concerning mutational landscape and expression of SUZ12 mRNA in HNSCC were retrieved from three publicly available databases including cBioPortal (http://www.cbioportal.org/),^23^ Oncomine (https://www.oncomine.org/)^24^ and TCGA (https://cancergenome.nih.gov/). The expression levels of SUZ12 mRNA (log2‐transformed) in HNSCC and normal counterparts were retrieved and statistically compared. The associations between expression status of SUZ12 (high or low using median value as cut‐off) and patient survival were determined by Kaplan‐Meier analysis (log‐rank test). Correlation analysis of SUZ12 was performed in gene expression profiles available in the TCGA data set with MATLAB software (https://cn.mathworks.com). To identify potential biological processes and the Kyoto encyclopaedia of genes and genomes (KEGG) signalling pathways associated with SUZ12 expression in HNSCC, the candidate genes which were positively and negatively correlated with SUZ12 in TCGA‐HNSCC samples were identified extracted and further analysed using the DAVID web tool (http://david.abcc.ncifcrf.gov/home.jsp).

### 2.9 HNSCC patients and tissue samples

A retrospective cohort of 201 patients with primary HNSCC treated at our institution between January 2005 and December 2012 were enrolled. Written informed consent was obtained from these patients before study. Patient inclusion criteria were described as follows: primary HNSCC without any prior history of surgery, chemotherapy or radiotherapy; patients underwent radical tumour resection and neck dissection (elective or therapeutic neck dissection as required); detailed information available including epidemiologic, clinical, pathological and follow‐up data. The archived tissue samples were collected, and the haematoxylin‐eosin staining slides for all patients were further examined to confirm the previous diagnose based on the established histopathological criteria. In addition, 20 pairs of fresh HNSCC samples and adjacent non‐cancer tissue were collected upon cancer resection and subjected to Western blot assay for SUZ12 protein measurement. This study protocol was reviewed and approved by the Research Ethic Committee of Nanjing Medical University (2016‐0516).

### 2.10 Histopathological evaluation, clinicopathological categorization and immunohistochemical staining of SUZ12 in HNSCC samples

All relevant clinicopathological parameters for each patient including histological grade, TNM classification and clinical stage were determined similarly as we described before.^12^, ^25^ Immunohistochemical staining for SUZ12 was performed on 4‐μm formalin‐fixed, paraffin‐embedded specimens. In brief, tissue sections from representative paraffin blocks were deparaffinized in xylene and rehydrated through graded alcohols. Tissue slides were then processed in microwave heating in citrate buffer (10 mmol/L, pH 6.0, 15 min) for antigen retrieval and hydrogen peroxide (3%) for endogenous peroxidase inactivation. These sections were further incubated with primary antibody (anti‐SUZ12, 1:200 dilution; Abcam, ab112073, USA) at 4°C overnight and developed with 3,3′‐diaminobenzidine and counterstained with haematoxylin. The immunoreactivity in each slide was assessed independently by two senior pathologists who were blinded to the relevant clinicopathological data. Negative controls (without primary antibody incubation) were included in each staining run. Immunoreactivity was semiquantitatively evaluated based on staining intensity and distribution using the immunoreactive score which was calculated as intensity score × proportion score as we reported previously.^9^, ^26^ Intensity score was defined as 0, negative; 1, weak; 2, moderate; 3, strong. The proportion score was defined as 0, negative; 1, <10%; 2, 11%‐50%; 3, 51%‐80%; 4, >80% positive cells. Therefore, the total score ranged from 0 to 12. Accordingly, the immunoreactivity of each slide was categorized into three subgroups based on the final score: 0, negative; 1‐4, low expression; 4‐12, high expression, similar as our previous reports.^9^, ^25^, ^26^

### 2.11 Statistical analysis

All quantitative data reported here were shown as mean ± SD from two or three independent experiments as indicated and compared with Student's *t* test or ANOVA with Bonferroni post hoc test unless otherwise specified. The potential associations between SUZ12 expression and various clinicopathological parameters were evaluated by chi‐square or Fisher's exact test. The survival rates of patients were estimated using Kaplan‐Meier method and compared with log‐rank test. The prognostic analyses were performed by univariate and multivariate Cox regression models to determine the individual clinicopathological variables with patient overall survival. *P* values <.05 (two‐sided) were considered statistically significant. All statistical analyses were performed with GraphPad Prism 6 (GraphPad) or SPSS 21.0 software (IBM).

## 3 RESULTS

### 3.1 SUZ12 mRNA is frequently overexpressed in HNSCC as determined via bioinformatics analyses

**Figure 1:**
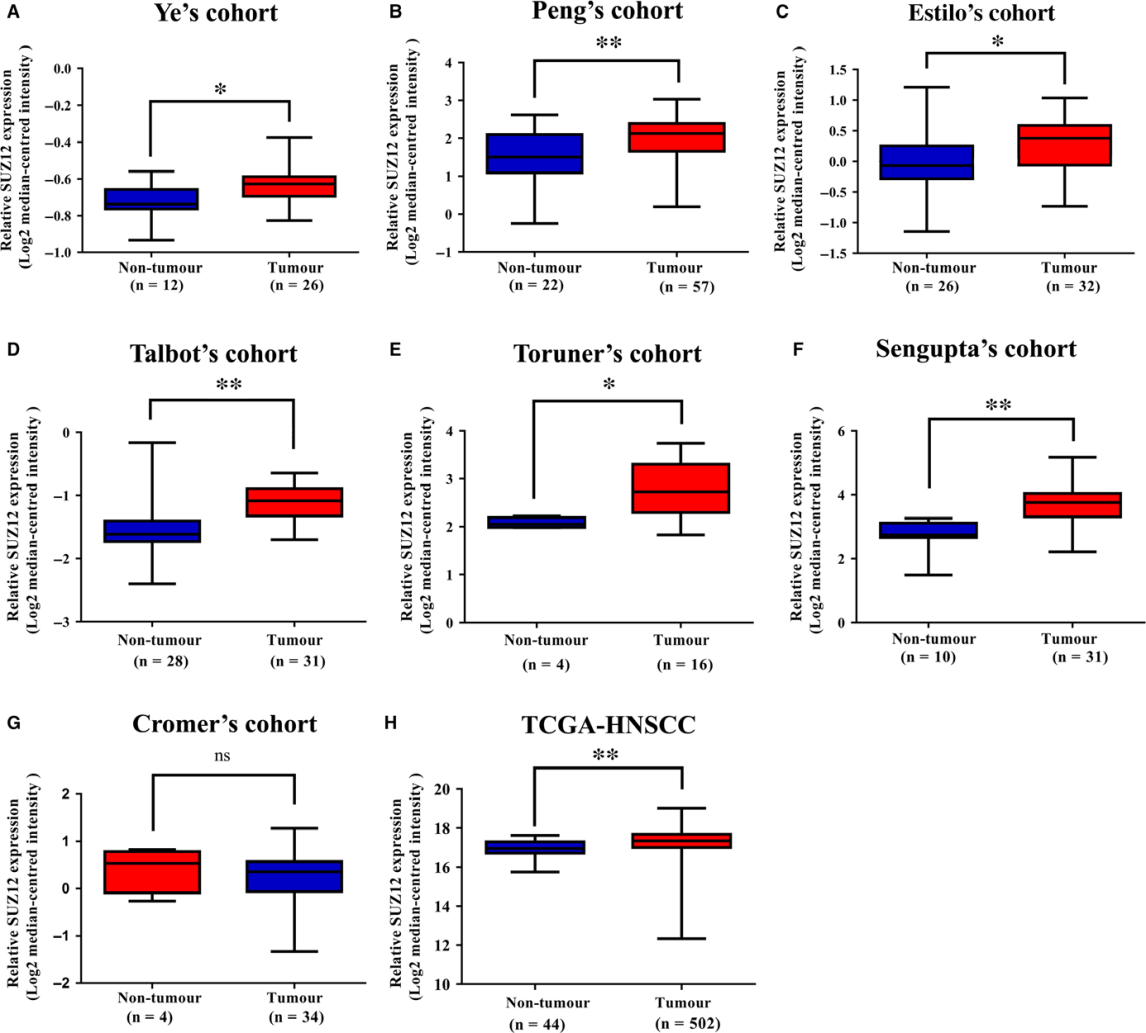
Overexpression of SUZ12 mRNA in multiple HNSCC cohorts. The mRNA levels of SUZ12 (log2‐transformed) were compared between HNSCC samples and normal counterparts in multiple patient cohorts. (A‐H) The original data were retrieved from Oncomine database and TCGA and then plotted using GraphPad Prism 6.0 software. *Y*‐axis represents the median intensity, 25th and 75th percentile data. *P <* *.05; **P <* *.01; ns, not significant; Student's *t* test or Mann‐Whitney U test as appropriate

Accumulating evidence has indicated that SUZ12 is usually up‐regulated in multiple cancers and associates with unfavourable prognosis.^14^, ^18^, ^25^ Initially, to explore the mutational landscape and mRNA expression of SUZ12 in HNSCC, we utilized three publicly available databases including cBioPortal, Oncomine and TCGA and analysed the relevant data. As shown in Figure 1A‐F, data mining and interrogation from Oncomine database indicated significant higher expression of SUZ12 mRNA in HNSCC samples from several independent patient cohorts as compared to normal counterparts, respectively, except the Cromer's cohort.^27^, ^28^, ^29^, ^30^, ^31^, ^32^, ^33^ Data integration and analyses from TCGA‐HNSCC cohort (502 cases) using cBioPortal platform indicated that total frequency of SUZ12 genetic alterations in HNSCC samples was less than 2.5%. Moreover, SUZ12 mRNA is significantly overexpressed in TCGA‐HNSCC samples as compared to non‐tumour samples (44 cases) (Figure. 1H). To uncover potential associations between SUZ12 mRNA expression status and the clinicopathological parameters, we compared its abundance among diverse groups based on pathological grade and clinical stage, respectively. However, as shown in Figure S1A, B, the abundance of SUZ12 mRNA was comparable without significant difference among patient groups stratified by pathological grade and clinical stage. In addition, there was no significant association between SUZ12 mRNA expression and patient overall survival (Kaplan‐Meier analysis, log‐rank test, *P *=* *.773), when the median value of SUZ12 mRNA was employed as cut‐off to stratify patients into low or high SUZ12‐expressing groups (Figure S2).

### 3.2 SUZ12 protein is significantly up‐regulated and associates with cervical node metastasis in primary HNSCC samples

**Figure 2:**
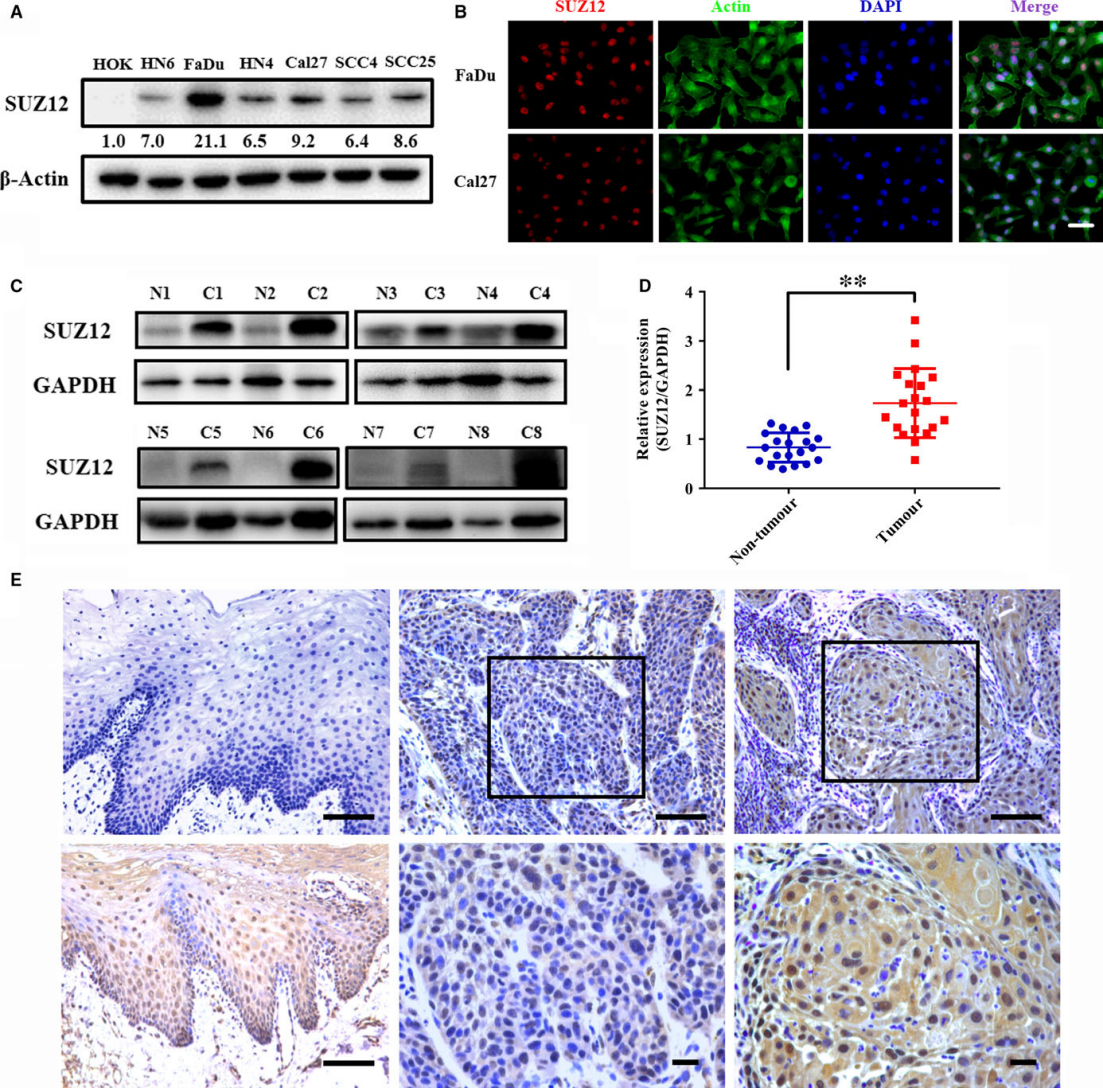
SUZ12 protein is overexpressed in HNSCC cell lines and primary samples. A, The abundance of SUZ12 protein in a panel of HNSCC cell lines was measured by Western blot and compared with non‐tumorigenic human oral keratinocytes (HOK). Representative images of Western blot were shown from three independent experiments. B, The intracellular localization of SUZ12 protein was visualized by immunofluorescent staining in two selected HNSCC cells FaDu and Cal27. Scale bar: 50 μm. C, D, The protein abundance of SUZ12 in 20 pairs of fresh HNSCC samples and adjacent non‐tumour epithelial was determined by Western blot. Representative images of Western blot were shown from 8 pairs of samples (C). The quantification data of SUZ12 protein were shown and statistically compared (D). **P <* *.01, Student's *t* test. E, Representative negative (left, upper) and positive (left lower) staining of SUZ12 in normal oral epithelial was shown in left panel. Representative low expression of SUZ12 in primary human HNSCC sample was shown in middle panel. The area marked by black box (middle, upper) was shown in larger magnification (middle, lower). Representative high expression of SUZ12 in primary human HNSCC sample was shown in right panel. The area marked by black box (right, upper) was shown in larger magnification (right, lower). Nuclei are counterstained with haematoxylin. Scale bar: 100 μm

To further determine the expression of SUZ12 in HNSCC samples, the abundance of SUZ12 protein in several HNSCC cell lines was measured. As displayed in Figure 2A, significant up‐regulation of SUZ12 protein was detected in all cancerous cell lines examined as compared to immortalized non‐tumorigenic cells HOK. Images from immunofluorescence assay showed clear nuclear enrichment of SUZ12 protein in two selected HNSCC cell lines FaDu and Cal27 (Figure 2B), which was consistent with its roles as a chromatin modifier. Moreover, the expression of SUZ12 protein was also determined in 20 pairs of HNSCC samples and adjacent non‐tumour tissue. As indicated in Figure 2C,D, marked overexpression of SUZ12 was observed in HNSCC relative to corresponding non‐tumour tissue (*P *< .001, Mann‐Whitney test). In agreement with this, the abundance of the epigenetic marker H3K27me3 as surrogate marker of SUZ12‐associated PRC2 activity was also significantly increased in HSNCC relative to normal counterparts (Figure S3). Furthermore, we semiquantitatively examined the expression pattern of SUZ12 protein by immunohistochemical staining in primary HNSCC samples from a retrospective cohort of 201 patients. All relevant information and clinicopathological parameters of these patients are listed in Table**** 1. In brief, 112 males and 89 females were enrolled with average age 64.5 years. The time durations for patient follow‐up ranged from 2 to 100 months (average 44.7 months).

**Table 1:** Associations between SUZ12 expression and multiple clinicopathological parameters in primary HNSCC

Clinicopathological parameters	Cases	SUZ12		*P*‐values
		Low	High	
Gender		89	112	
Male	112	53	59	.3913
Female	89	36	53	
Age				
≤60	92	36	56	.2008
>60	109	53	56	
Smoking				
No	170	80	90	.0773
Yes	31	9	22	
Alcohol use				
No	167	78	89	.1346
Yes	34	11	23	
Tumour size				
T1	89	42	47	.8500
T2	60	24	36	
T3	24	11	13	
T4	28	12	16	
Pathological grade				
I	113	48	65	.3464
II	47	25	22	
III	41	16	25	
Cervical node metastasis				
N(0)	133	66	67	**.0329**
N(+)	68	23	45	
Clinical stage				
I	45	23	22	.6030
II	60	28	32	
III	46	18	28	
IV	50	20	30	

The number in bold indicates statistical significance with *P*‐values <.05.

As shown in Figure 2E, SUZ12 positive staining was identified mainly in nucleus and scarcely in cytoplasm in HNSCC, whereas negative or weak staining was detected in the normal counterparts (20 oral mucosae obtained from non‐tumour surgery and histologically verified) as well as tumour stroma in HNSCC samples. According to our immunohistochemistry scoring method, SUZ12 protein abundance in these HNSCC and normal oral mucosa was further categorized. SUZ12 expression in HNSCC samples was categorized as low (89) or high (112), while negative (8), low (9) and high (3) in normal samples, thus indicating significant overexpression of SUZ12 protein in HNSCC (*P *< .0001, chi‐square test).

### 3.3 SUZ12 overexpression positively associates with cervical node metastasis and overall survival

The detailed correlations between SUZ12 expression and the clinicopathological parameters in HNSCC are analysed and shown in Table 1. No significant correlations between SUZ12 and patient age, gender, smoking and alcohol drinking, pathological grade, tumour size as well as clinical stage were found. Notably, SUZ12 expression was found to be positively associated with the presence of cervical lymph nodes metastasis (*P *=* *.0329, Fisher's exact test).

**Figure 3:**
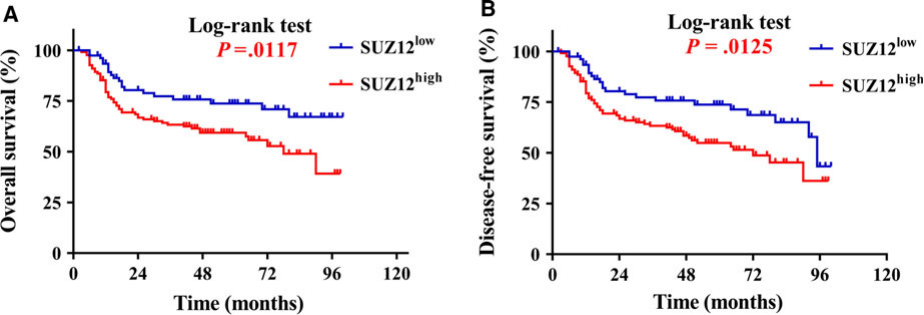
High SUZ12 expression positively associates with reduced overall and disease‐free survival in HNSCC patients. Overall (A) and disease‐free (B) survival analyses of patients with high or low expression of SUZ12 were estimated by Kaplan‐Meier method and compared with log‐rank test

To probe prognostic value of SUZ12 expression for patients with HNSCC, we next aimed to determine possible relationship between its overexpression and clinical outcome. Until the last follow‐up, 121 (60.2%) patients remained alive with disease‐free, 8 (4.0%) still alive but with recurrences and/or cervical nodal metastases, 72 (35.8%) died due to post‐surgical recurrence, metastases or other diseases. The Kaplan‐Meier survival analyses revealed that high SUZ12 expression in HNSCC significantly associated with shorter overall survival and disease‐free survival in comparison with its low counterparts (log‐rank test, *P *=* *.0117, .0125, Figure 3).

### 3.4 SUZ12 is involved in chemical‐induced HNSCC tumorigenesis

**Figure 4:**
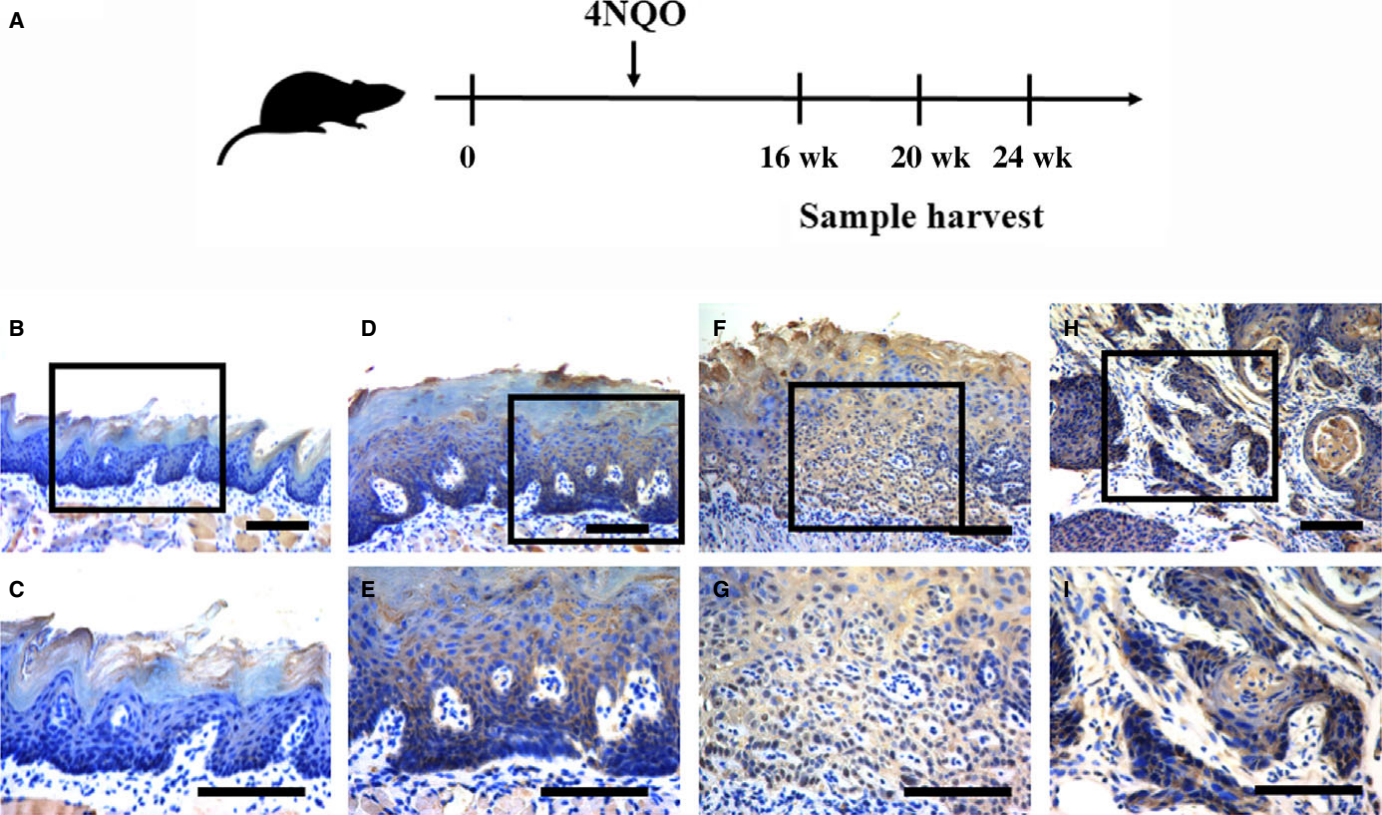
SUZ12 expression during HNSCC tumorigenesis in 4NQO‐induced animal model. A, Experimental scheme of 4NQO‐induced HNSCC animal model. B‐I, Immunohistochemical staining of SUZ12 in samples from diverse stages in 4NQO‐induced animal model. Images in the upper panel (B, D, F, H) were representative staining of SUZ12 in normal, epithelial with hyperplasia, epithelial with severe dysplasia/carcinoma in situ and squamous cell carcinoma, respectively. Images in the lower panel (C, E, G, I) were magnified from the black box area in the B, D, F, H images in the upper panel, respectively. Scale bar: 100 μm

Having revealed aberrantly high expression of SUZ121 in human HNSCC samples, we next developed a well‐established chemical‐induced animal model to characterize the expression pattern of SUZ12 during HNSCC initiation and progression (Figure 4A). Pathological lesions were primarily identified in tongue following 4NQO treatment and displayed typical changes from epithelial hyperplasia, dysplasia, carcinoma in situ and invasive SCC, thus largely recapitulating the multiple‐staged tumorigenic process in human HNSCC. Furthermore, as shown in Figure 4B, immunohistochemical staining of SUZ12 in these samples indicated negative or low staining in normal tongue mucosa and epithelial with hyperplasia, while prominent strong nuclear staining in carcinoma in situ and invasive carcinoma. Data from immunohistochemical staining revealed that significant SUZ12 overexpression was observed in carcinoma (62.5%, 5/8), whereas much less were detected in healthy mucosa (16.7%, 1/6), samples with hyperplasia (16.7%, 1/6) or dysplasia/carcinoma in situ (50.0%, 3/6). Together, our findings from chemical‐induced animal model provide support to the notion that SUZ12 might be critically involved in HNSCC development probably by serving as a putative oncogene.

### 3.5 SUZ12 knock‐down inhibits proliferation, migration and invasion in HNSCC cells

**Figure 5:**
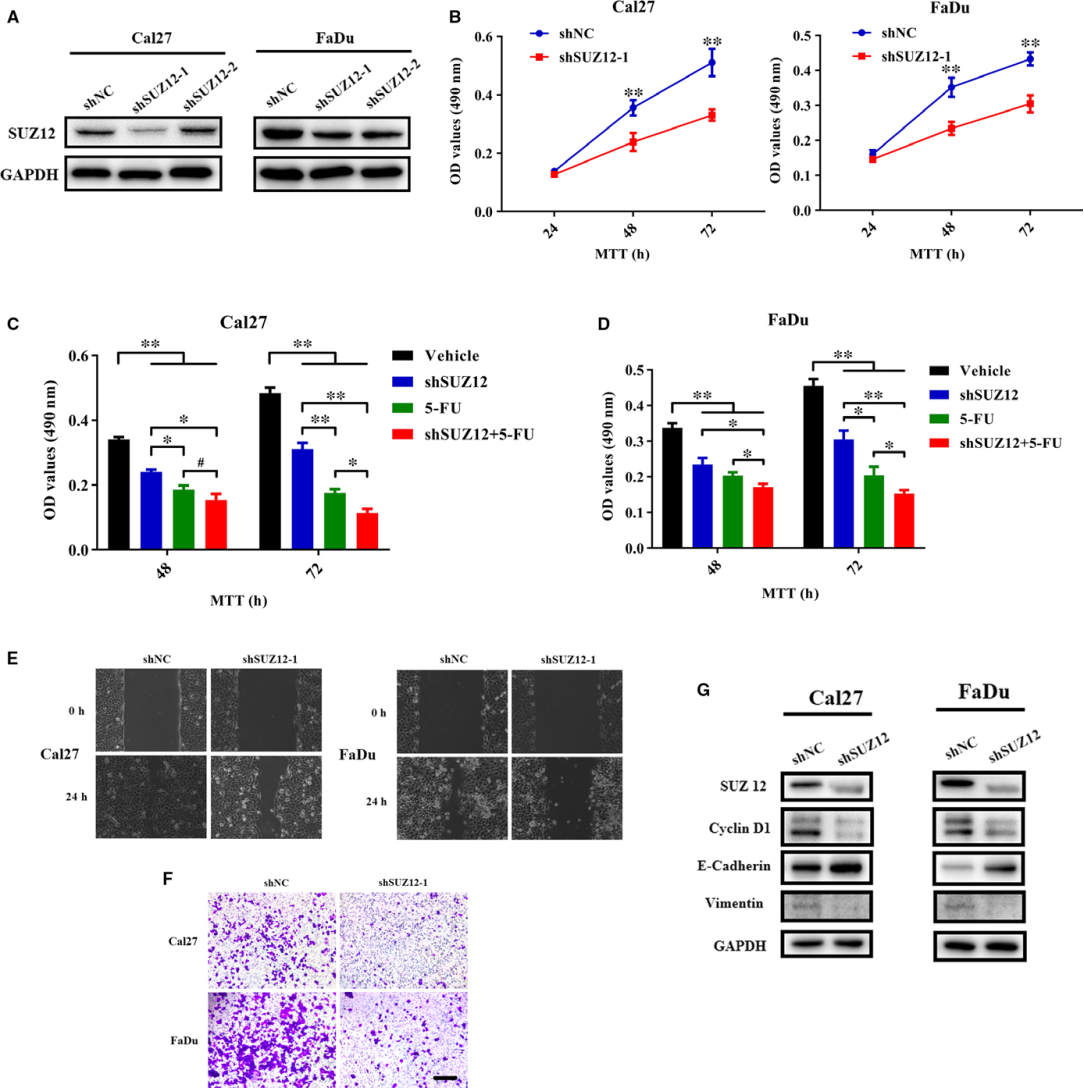
SUZ12 knock‐down resulted in impaired cell proliferation, migration and invasion, while enhanced chemosensitivity to 5‐FU. A, The knock‐down efficiency of two shRNA lentiviral vectors targeting human SUZ12 was measured by Western blot. The shSUZ12‐1 with higher knock‐down potency was selected for the following experiments. Representative images of Western blot were shown from three independent experiments. B, Cell proliferation was determined after Cal27 and FaDu cells were infected with shSUZ12‐1 lentivirus by MTT assay. C, D, Cell viability was probed in Cal27 (C) and FaDu (D) cells treated with shSUZ12‐1 lentivirus and 5‐FU alone or in combination for 48 h by MTT assay. E, Cell migration was determined in cells with stable SUZ12 knock‐down by wound‐healing assay. F, Cell invasion was determined in cells with stable SUZ12 knock‐down by transwell invasion assay. Scale bar: 100 μm. G, The abundance of proliferative marker cyclin D1 and migration/invasion‐relevant marker E‐cadherin and Vimentin was compared in cells infected shSUZ12 lentivirus or control virus. Representative images of Western blot were shown from three independent experiments. ^#^ *P *>* *.05, *P <* *.05, **P <* *.01, Student's *t* test

Having revealed the overexpression of SUZ12 and its clinical significance in HNSCC, we next sought to dissect its oncogenic roles driving HNSCC initiation and progression by shRNA‐mediated loss‐of‐function approach. We designed and synthesized two independent shRNA sequences targeting human SUZ12 mRNA which were cloned into lentiviral vectors, sequenced and packaged. We selected two HNSCC cell lines with relatively high endogenous SUZ12 (Fadu and Cal27) and introduced these lentiviral vector targeting SUZ12 (shSUZ12‐1, shSUZ12‐2) into them and then monitored the phenotypical changes in vitro. As shown in Figure 5A, SUZ12 protein was significantly reduced upon shSUZ12 lentivirus infection in both cells. Stable knock‐down cells with shSUZ12‐1 follow antibiotics selection were utilized for subsequent experiments. Results from MTT assay indicated that cell proliferation was potently inhibited in both cells upon SUZ12 knock‐down (Figure 5B). Interestingly, we treated stable shSUZ12 cells with 5‐FU, a common chemotherapeutic agent for HNSCC and found that cells with SUZ12 silencing had enhanced chemosensitivity to 5‐FU as evidenced by significant lower OD values in shSUZ12 cell treated with 5‐FU as compared with shSUZ12 cells or cells treated with 5‐FU alone (Figure 5C,D). However, the ratios of cells undergoing apoptosis upon SUZ12 knock‐down were comparable to control cells without significant difference (data not shown). Moreover, the migratory and invasive potentials of cell following SUZ12 knock‐down were also measured using wound‐healing and transwell assays, respectively. As shown in Figure 5E,F, SUZ12 knock‐down significantly reduced both migratory and invasive properties of cells in vitro. In agreement with these observed phenotypical changes following SUZ12 depletion, the expression of cell proliferation marker cyclin D1 was markedly reduced in SUZ12 knock‐down cells, while the EMT/metastasis‐associated marker Vimentin was down‐regulated concomitant with E‐cadherin up‐regulation (Figure 5G).

**Figure 6:**
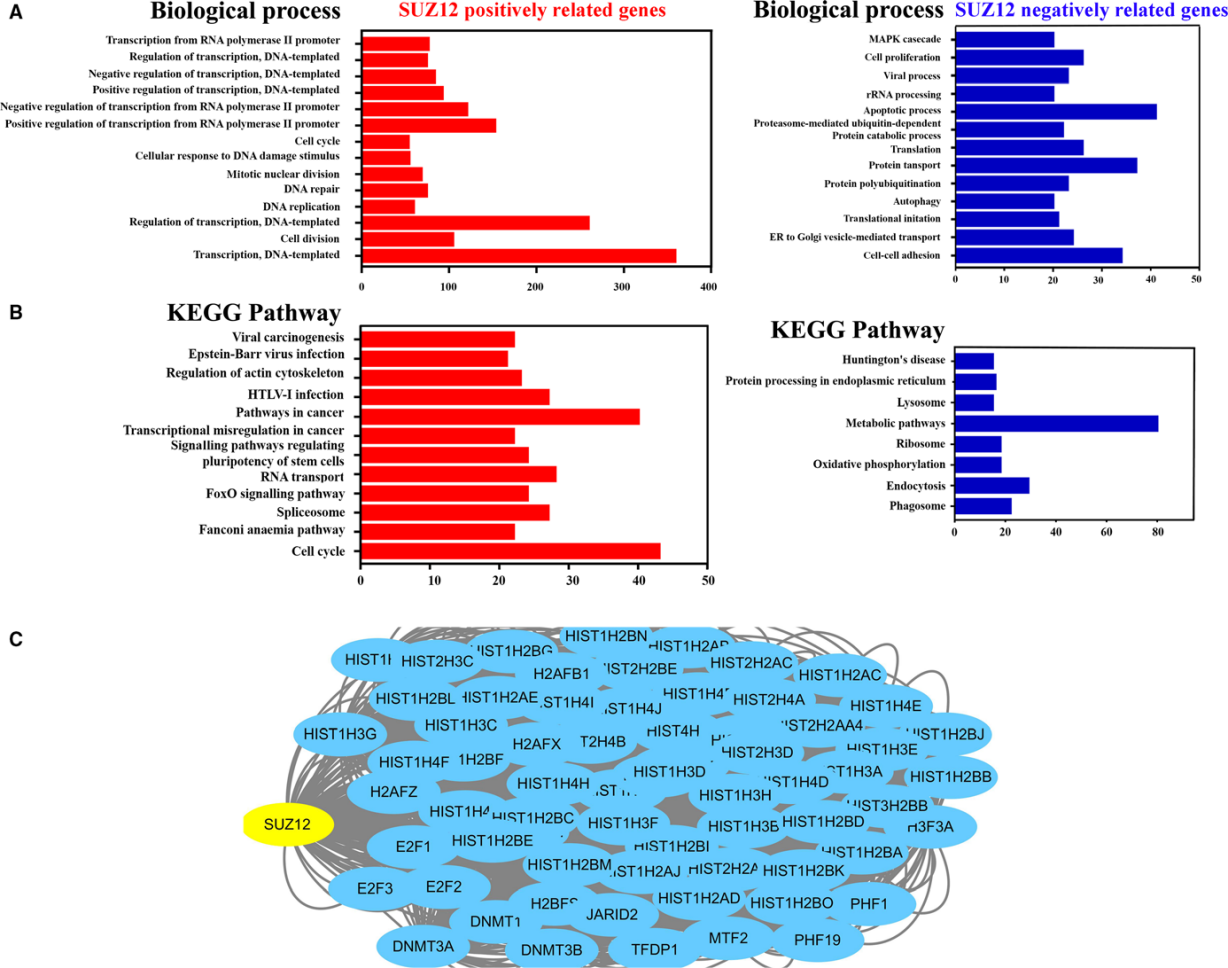
Gene ontology and KEGG pathway analyses of SUZ12‐related genes in HNSCC. A, GO biological process analyses of SUZ12 positively and negatively related genes identified from TCGA‐HNSCC database. B, KEGG pathway analyses of SUZ12 positively and negatively related genes identified from TCGA‐HNSCC database. C, Network formed by SUZ12 and its most frequently altered neighbour genes (50 of a total of 69) produced by cBioPortal platform

To complement the in vitro loss‐of‐function assay in exploring pro‐tumorigenic functions of SUZ12, we further performed bioinformatics analyses using TCGA‐HNSCC data to identify the candidate genes whose expression was potentially correlated with SUZ12 which were subjected to gene ontology (GO) and pathway analyses. A total number of 2606 SUZ12‐related genes (1762 positive‐related, 864 negative‐related) in TCGA‐HNSCC data were identified and subsequently subjected to GO/pathway analyses. As shown in Figure 6A, GO analysis indicated that genes positively associated with SUZ12 were significantly enriched in cell division, cell cycle, mitotic nuclear division and transcription regulation. On the contrary, the genes negatively related with SUZ12 were involved in apoptotic process, cell‐cell adhesion, etc. Furthermore, KEGG pathway analyses indicated those SUZ12 positively related genes were significantly enriched in cell cycle, pathways in cancer, transcriptional misregulation in cancer and viral carcinogenesis, etc. (Figure 6B). In addition, as shown in Figure 6C, analyses through cBioPortal platform revealed interactive network containing 51 nodes including SUZ12 and the 50 most frequently altered neighbour genes in HNSCC. Consistent with its primary roles as a chromatin modifier, most altered neighbours of SUZ12 were histone‐related genes and PRC components. Notably, some well‐established oncogenes such as DNMT1, 3 as well as JARID2 were also identified. Taken together, our in vitro cellular assay and bioinformatics assay both strongly favour the notion that SUZ12 is a novel putative oncogene in HNSCC.

### 3.6 SUZ12 knock‐down inhibits tumour growth in a HNSCC xenograft model

**Figure 7:**
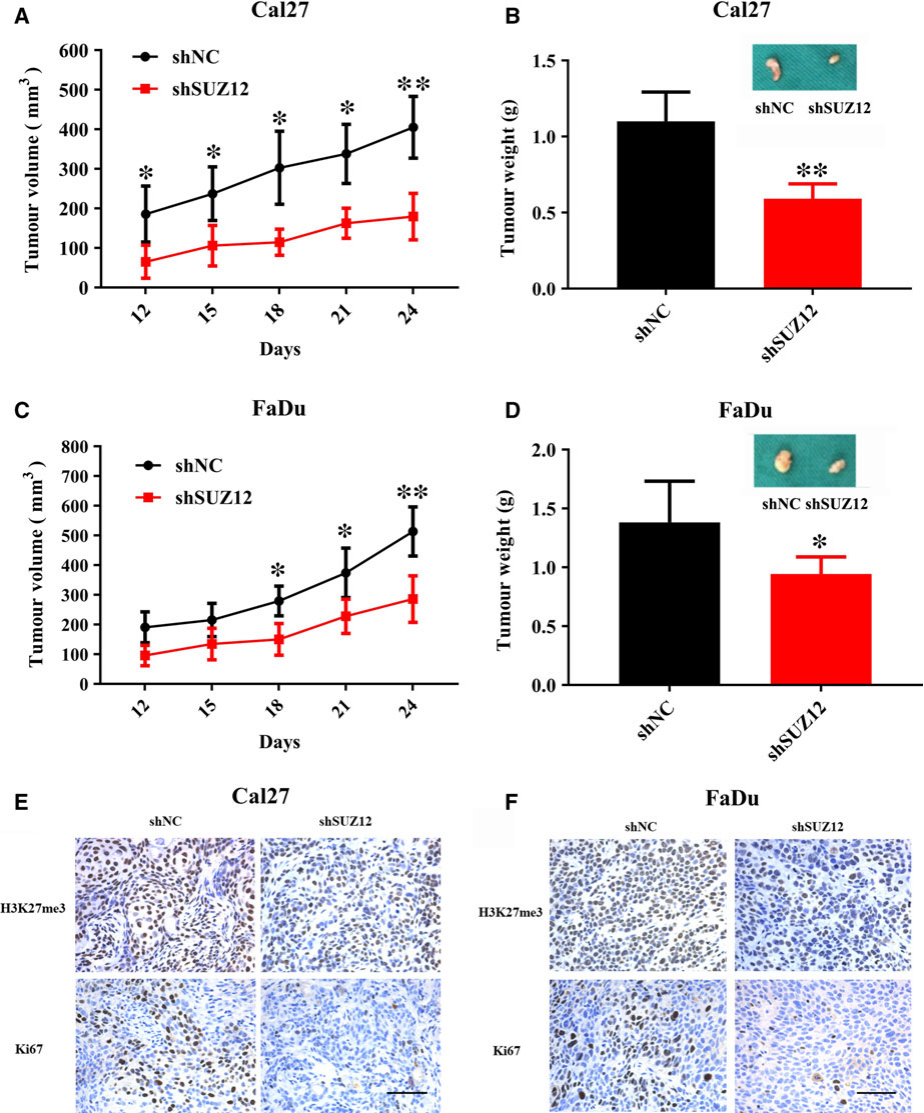
SUZ12 depletion impaired tumour growth in a HNSCC xenograft model. A, Tumour volume was monitored in xenograft samples derived from Cal27 cells with stable SUZ12 knock‐down or controls. B, Final weight of tumour masses harvested from derived from Cal27 cells with stable SUZ12 knock‐down or controls was compared. C, Tumour volume was monitored in xenograft samples derived from FaDu cells with stable SUZ12 knock‐down or controls. D, Final weight of tumour masses harvested from derived from FaDu cells with stable SUZ12 knock‐down or controls was compared. E, F, The marker indicative of SUZ12 knock‐down H3K27me3 and proliferative marker Ki67 was determined by immunohistochemical staining in xenograft samples derived from Cal27 (E) and FaDu (F) cells with stable SUZ12 knock‐down or controls. Scale bar: 100 μm. Representative images are shown. *P <* *.05, **P <* *.01, Student's *t* test

To substantiate the oncogenic roles of SUZ12 in HNSCC, we developed a HNSCC xenograft model in which stable SUZ12 knock‐down cells were inoculated into left flanks of nude mice. Then, tumour incidence and growth were monitored after cell inoculation. As shown in Figure 7A‐D, tumour growth was compromised in xenograft samples formed from SUZ12‐silencing cells as compared to those from control cells as evidenced by markedly reduced tumour volume and weight. Nevertheless, the incidence of tumour masses formed was comparable in SUZ12 knock‐down cells and control cells at two weeks after cell transplantation. Immunohistochemical staining of tumour samples revealed significantly reduced H3K27me3 staining, a surrogate marker of SUZ12 expression and function, in samples from SUZ12 knock‐down cells in comparisons with controls (Figure 7E,F). Moreover, the number of Ki67‐positive cells was significantly reduced in tumour samples from SUZ12 knock‐down cells as compared to samples formed from control cells (Figure 7E,F). Together, these findings revealed that SUZ12 knock‐down impaired tumour growth of HNSCC in vivo, suggesting that SUZ12 might be required for HNSCC growth.

## 4 DISCUSSION

The epigenetic modifying complexes PRC1 and PRC2 regulate downstream targets by regulating chromatin structure and have been critically involved in multiple physiological and pathological processes including stem cell self‐renewal and differentiation, cell apoptosis as well as tumorigenesis.^7^, ^34^ Previous studies have suggested that SUZ12 might be a putative oncogene involving tumorigenesis and serve as a potential diagnostic biomarker as well as anticancer therapeutic target.^14^, ^15^, ^17^, ^35^ Herein, we revealed the expression pattern of SUZ12 in HNSCC, determined its clinicopathological and prognostic significance and also uncovered its pro‐tumorigenic roles by loss‐of‐function approach. Our findings together indicate that SUZ12 serves as a novel putative oncogene to promote HNSCC tumorigenesis and also a new biomarker with translational potentials.

HNSCC initiation and progression are characterized by multiple stages from normal epithelial to SCC driven by genetic predisposition, activation of oncogenes and inactivation of tumour suppressor genes.^4^ Particularly, epigenetic modifications underlying oncogene or tumour suppressor dysregulations have been found to contribute to almost all stages during HNSCC tumorigenesis.^36^ Among these cancer epigenetic modulators, PRC1/2 has been increasingly recognized as key mediators to facilitate cancer initiation, unchecked growth and metastatic dissemination, and also has been demonstrated as therapeutic targets with considerable promise.^7^, ^34^ Here, we found that SUZ12 is aberrantly up‐regulated in a large fraction of HNSCC samples as evidenced by significantly elevated SUZ12 mRNA in multiple cancer clinical data sets as well as overexpression of SUZ12 protein in our patient cohort. This is consistent with previous findings which revealed elevated SUZ12 in gastric, bladder, lung and colorectal cancer.^14^, ^18^, ^19^, ^35^ Moreover, our findings from 4NQO‐induced animal model reveal that increased SUZ12 expression was observed during disease progression from hyperplasia to invasive carcinoma, thus giving further support to the idea that SUZ12 as a bona fide oncogene promotes tumorigenesis in multiple contexts including HNSCC. To the best of our knowledge, this might be the first study to reveal the abnormal overexpression pattern of SUZ12 in HNSCC.

Previous studies have revealed important clinical relevance of SUZ12 overexpression in human cancer.^14^, ^17^, ^25^ For example, elevated SUZ12 expression significantly associated with large tumour size, lymph node metastasis and advanced stages in non‐small cell lung cancer and colorectal cancer.^17^, ^18^ Having established overexpression pattern of SUZ12 in HNSCC, we further revealed that its up‐regulation significantly associated with cervical node metastasis, while the correlations between other clinicopathological parameters and SUZ12 expression did not reach statistical significance. Moreover, results from Kaplan‐Meier survival analyses revealed that SUZ12 up‐regulation was significantly associated with reduced survival, thus suggesting that SUZ12 might be promising prognostic biomarkers for HNSCC. However, we failed to identified SUZ12 expression as an independent prognostic predictor for HNSCC by performing multivariate survival analyses (Cox proportional hazards regression model, data not shown), although previous studies have found that elevated SUZ12 expression associates with adverse prognosis and serves as an independent prognostic predictor for patients with some types of cancer.^14^, ^19^, ^25^ We reasoned that this discrepancy might be caused by sample size, retrospective nature of our study, patient heterogeneity, and different regime for IHC scoring and patient stratification. Thus, more clinical studies are needed to further substantiate the prognostic value of SUZ12 in HNSCC.

Accumulating evidence has indicated that SUZ12 is critically involved in tumorigenesis by promoting cell proliferation, migration and suppressing apoptosis.^14^, ^15^, ^17^, ^19^ In line with this, our findings from in vitro loss‐of‐function assay reveal that loss of SUZ12 resulted in reduced proliferation, migration and invasion in HNSCC cells. These findings were also further substantiated by the facts such as reduced xenograft tumour growth upon SUZ12 depletion and positive association between SUZ expression and cervical node metastasis in patient cohort. Consistently, previous studies have revealed that loss of miR‐200b in breast cancer increased SUZ12 expression and H3K27me3 abundance, in turn, resulted in repression of E‐cadherin and impaired invasiveness and metastasis.^15^ Complementary, Herranz N and his colleagues revealed that the EMT master factor Snail interacted with SUZ12 and recruited PRC2 to the E‐cadherin promoter, which in turn repressed E‐cadherin expression and enhanced cancer metastasis.^37^ In bladder cancer, SUZ12 was critically involved in TGF‐β1‐induced up‐regulation of malat1, EMT and metastasis.^38^ Of note, pharmacological or genetic depletion of SUZ12 inhibited cell proliferation, impaired the migratory and invasive properties and disrupted the maintenance of cancer stem cell, ultimately induced tumour regression and reduced tumour metastasis.^15^, ^17^, ^35^, ^39^ Together, our findings together with others strongly suggest that SUZ12 probably functions as a putative pro‐tumorigenic gene via enhancing cancer cell proliferation and invasion. Selective targeting of SUZ12 by genetic or pharmacological approach might hold translational promise against HNSCC.

In conclusion, our findings reveal aberrantly overexpressed SUZ12 in a significant subset of HNSCC and unravel its oncogenic roles to promote initiation and progression of HNSCC. Large knowledge gap still remains between the functional roles of SUZ12 during HNSCC tumorigenesis and efficient therapeutic approaches to targeting SUZ12.

## CONFLICT OF INTEREST

The authors declare that they have no competing interests.

## AUTHOR CONTRIBUTIONS

Drs.Yaping Wu, Huijun Hu and Wei Zhang performed the experiments, data collection, statistical analyses and drafted the manuscript. Drs. Zhongwu Li, Pengfei Diao, Dongmiao Wang, Wei Zhang, Yanling Wang, Jianrong Yang participated the study design, data collection and analyses. Dr.Jie Cheng conceived and supervised the whole project and drafted the manuscript. All authors read and approved the final manuscript.
