# Supplementary figures and images for: SUZ12 is a novel putative oncogene promoting tumorigenesis in head and neck squamous cell carcinoma
Source: J Cell Mol Med. 2018 Apr 18;22(7):3582–94. doi: 10.1111/jcmm.13638 (PMC6010759; doi:10.1111/jcmm.13638)

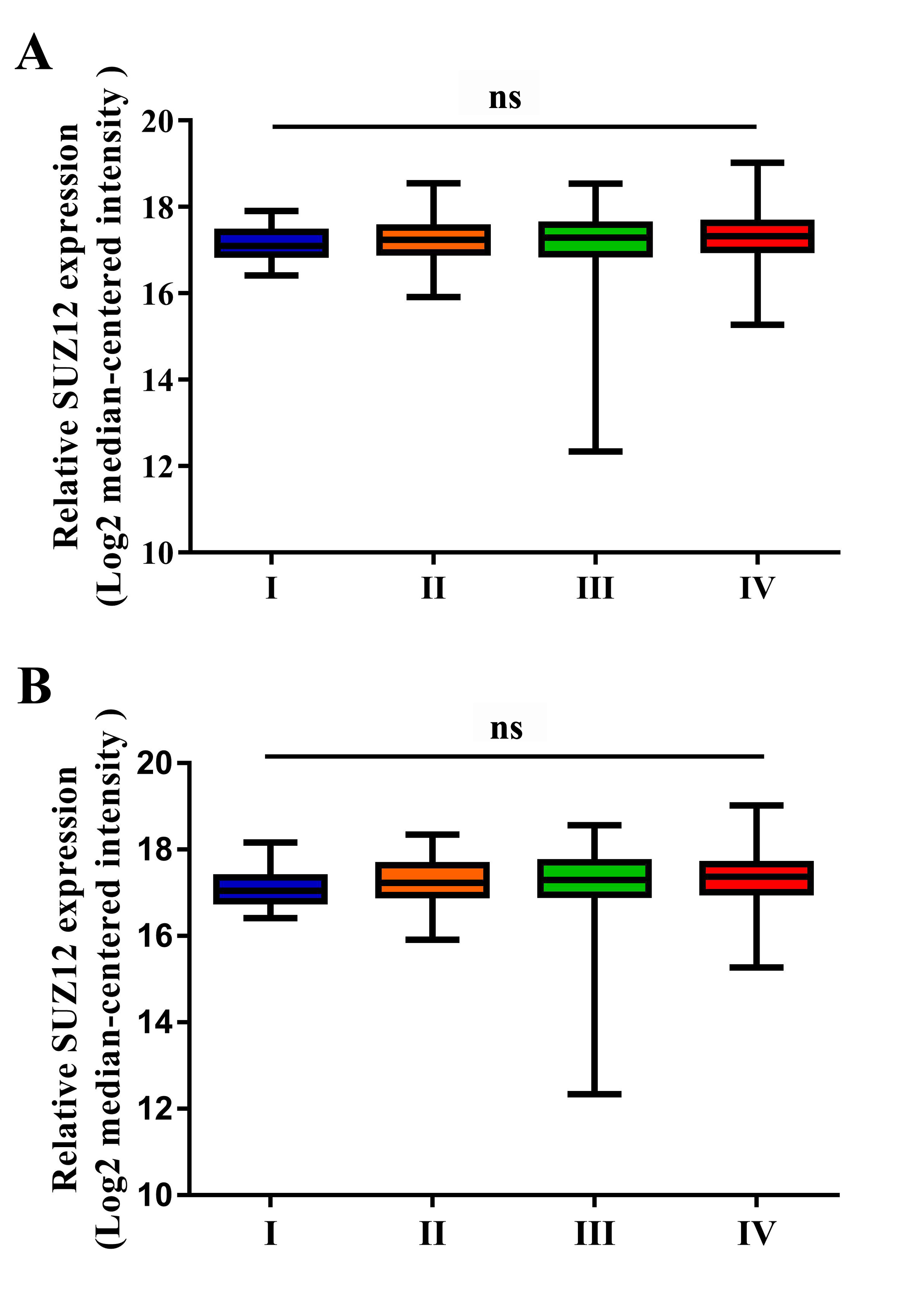

Supplement: Supplementary file 1 [file JCMM-22-3582-s001.tif]

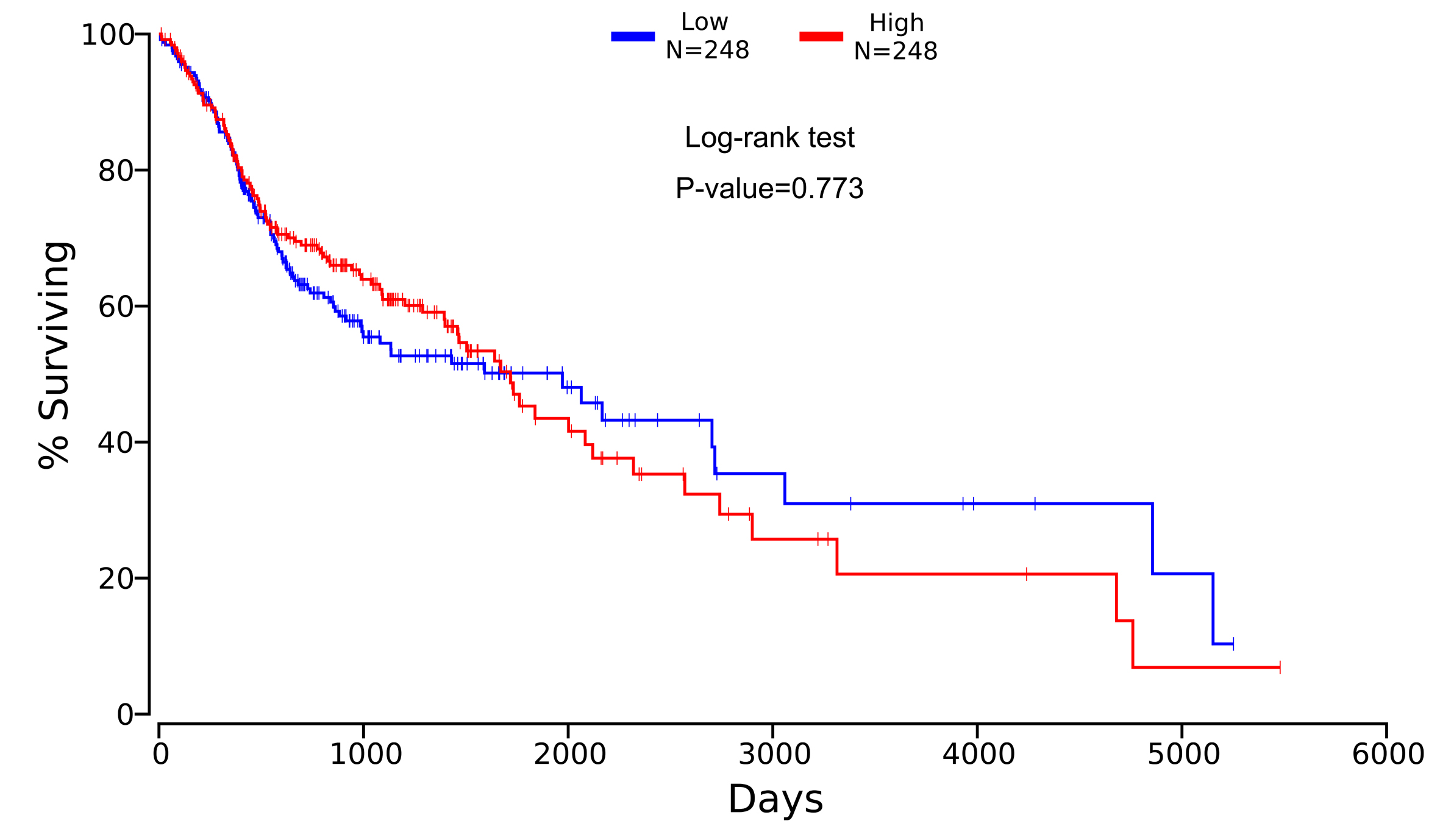

Supplement: Supplementary file 2 [file JCMM-22-3582-s002.tif]

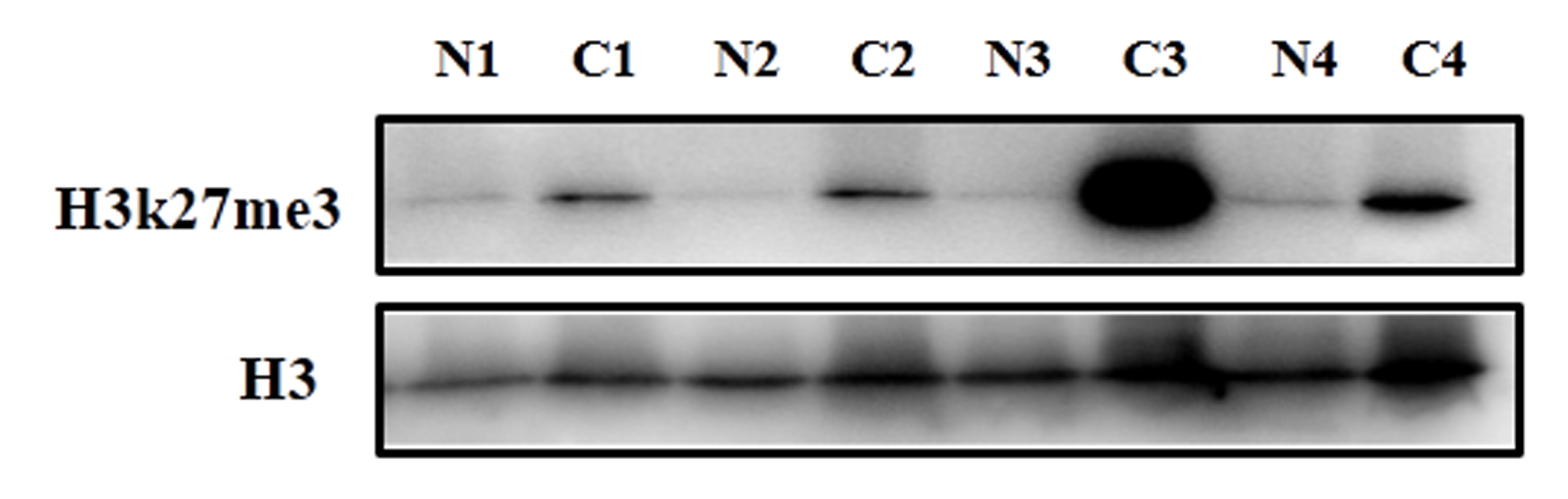

Supplement: Supplementary file 3 [file JCMM-22-3582-s003.tif]
